# Features of chaotic activity in a balanced network of Type II neuronal oscillators

**DOI:** 10.1186/1471-2202-13-S1-P10

**Published:** 2012-07-16

**Authors:** Maximilian Puelma Touzel, Michael Monteforte, Fred Wolf

**Affiliations:** 1 Max Planck Institute for Dynamics and Self-Organization, Goettingen, Germany; 2 Faculty of Physics, Georg-August-University, Goettingen, Germany; 3 Bernstein Center for Computational Neuroscience, Goettingen, Germany

## 

The taxonomy of collective states in models of neuronal networks must grow in tandem with relevant measures of their function to progress our understanding. One network state has emerged as an explanation for why many real neuronal networks display irregular, asynchronous network activity, despite being composed of reliable units that each process many inputs. This robust ‘balanced state’ attractor arises when the sum of the mean excitatory and mean inhibitory currents to each neuron is on average balanced and subthreshold, leaving fluctuations to drive spiking[1]. Chaos in a network’s dynamics, or lack thereof, is a useful functional measure because it can strongly constrain how the network will process its inputs[2]. Rates of information loss due to chaotic dynamics are connected to the stability metric known as the Lyapunov spectrum. Methods for its calculation have only recently appeared[3], where the application furthered our understanding of chaos in balanced networks. This and previous work have focused on cortical network models of neurons with integrator (Type I) excitability. Here, we extend this research to neurons with resonator (Type II) excitability. These include the mitral cells of the olfactory bulb, which exhibit intrinsic subthreshold membrane potential oscillations whose frequency matches that of spiking[4]. The mitral cell network displays irregular, yet precisely-timed activity. It also receives excitatory sensory activity from the olfactory mucosa and potentially balancing inhibitory recurrent input. We have performed the first Lyapunov analyses of a balanced resonator network, whose stability properties we seek to understand, and which we believe will help explain the role of olfactory bulb collective oscillations in behavioural discriminations[5].

We study a generic 2-dimensional linear threshold neuron model, which produces leaky integrator or resonator [6] dynamics, depending on the parameters.

The terms: a leakage term, a scalable coupling term, an input term from recurrent input, *X* , distributed across *V* and *W* according to *δ*, *γ∈* [0,1], and finally external driving term, which determines to where the state is attracted. *V* is a somatic voltage variable whose membrane time constant sets the units of time. W is an auxiliary current variable that takes on different biologically meaningful roles depending on the parameter values. We consider δ-pulse coupling with coupling strength matrix, *J_ij_*. For evolution between spikes, we have obtained the analytic solution to this model and implemented an efficient and precise root finding algorithm to obtain the next threshold-crossing time. Using this, we can perform numerically exact, event-based network simulations, iterating from one spike in the network to the next. We also analytically calculated the single spike Jacobian of this iterative map that describes how perturbations evolve between spikes and which we use to compute the full Lyapunov spectrum. We first have reproduced results from a separate implementation of the correlated leaky integrate-and-fire neuron. Then, by progressively increasing the frequency of subthreshold oscillations from 0, we will present our results on how the Lyapunov spectrum of the network changes across the transition from Type I to Type II neuron dynamics.
